# Evolution of Bacterial Global Modulators: Role of a Novel H-NS Paralogue in the Enteroaggregative *Escherichia coli* Strain 042

**DOI:** 10.1128/mSystems.00220-17

**Published:** 2018-03-20

**Authors:** A. Prieto, M. Bernabeu, S. Aznar, S. Ruiz-Cruz, A. Bravo, M. H. Queiroz, A. Juárez

**Affiliations:** a Department of Genetics, Microbiology and Statistics, University of Barcelona, Barcelona, Spain; b Centro de Investigaciones Biológicas, Consejo Superior de Investigaciones Científicas, Madrid, Spain; c Institute for Bioengineering of Catalonia (IBEC), The Barcelona Institute of Science and Technology, Barcelona, Spain; Leiden University

**Keywords:** EAEC, H-NS, gene regulation

## Abstract

Bacterial genomes sometimes contain genes that code for homologues of global regulators, the function of which is unclear. In members of the family *Enterobacteriaceae*, cells express the global regulator H-NS and its paralogue StpA. In *Escherichia coli*, out of providing a molecular backup for H-NS, the role of StpA is poorly characterized. The enteroaggregative *E. coli* strain 042 carries, in addition to the *hns* and *stpA* genes, a third gene encoding an *hns* paralogue (*hns2*). We present in this paper information about its biological function. Transcriptomic analysis has shown that the H-NS2 protein targets a subset of the genes targeted by H-NS. Genes targeted by H-NS2 correspond mainly with horizontally transferred (HGT) genes and are also targeted by the Hha protein, a fine-tuner of H-NS activity. Compared with H-NS, H-NS2 expression levels are lower. In addition, H-NS2 expression exhibits specific features: it is sensitive to the growth temperature and to the nature of the culture medium. This novel H-NS paralogue is widespread within the *Enterobacteriaceae*.

**IMPORTANCE** Global regulators such as H-NS play key relevant roles enabling bacterial cells to adapt to a changing environment. H-NS modulates both core and horizontally transferred (HGT) genes, but the mechanism by which H-NS can differentially regulate these genes remains to be elucidated. There are several instances of bacterial cells carrying genes that encode homologues of the global regulators. The question is what the roles of these proteins are. We noticed that the enteroaggregative *E. coli* strain 042 carries a new hitherto uncharacterized copy of the *hns* gene. We decided to investigate why this pathogenic *E. coli* strain requires an extra H-NS paralogue, termed H-NS2. In our work, we show that H-NS2 displays specific expression and regulatory properties. H-NS2 targets a subset of H-NS-specific genes and may help to differentially modulate core and HGT genes by the H-NS cellular pool.

## Abstract (precis)

Global regulators such as H-NS play key relevant roles enabling bacterial cells to adapt to a changing environment. H-NS modulates both core and horizontally transferred (HGT) genes, but the mechanism by which H-NS can differentially regulate these genes remains to be elucidated. There are several instances of bacterial cells carrying genes that encode homologues of the global regulators. The question is what the roles of these proteins are. We noticed that the enteroaggregative *E. coli* strain 042 carries a new hitherto uncharacterized copy of the *hns* gene. We decided to investigate why this pathogenic *E. coli* strain requires an extra H-NS paralogue, termed H-NS2. In our work, we show that H-NS2 displays specific expression and regulatory properties. H-NS2 targets a subset of H-NS-specific genes and may help to differentially modulate core and HGT genes by the H-NS cellular pool.

## INTRODUCTION

Bacterial global regulators play key roles in facilitating bacterial cells to adapt to a constantly changing environment. Several global regulators belong to the category of the nucleoid-associated proteins. Many of these proteins present a dual role: they contribute to the organization of the chromosome and also regulate gene expression. A well-studied example is the H-NS protein, widespread in the gammaproteobacteria and best studied in *Escherichia coli* and *Salmonella*. Alleles of the *hns* gene were independently identified several years ago as controlling environmental regulation of gene expression in *E. coli* and *Salmonella* (1–3). H-NS is considered mainly a repressor of gene expression (4). The use of chromatin immunoprecipitation (ChIP) to determine the precise location of H-NS along the bacterial genome showed that H-NS targets AT-rich DNA stretches (5–9). Consequently, H-NS has been proposed to operate as a xenogeneic silencer, silencing horizontally acquired DNA (7). Unwanted expression of horizontally transferred (HGT) DNA would result in fitness costs. Recent reports have shown that H-NS binding to AT-rich sequences results mainly in suppression of transcription from intragenic promoters. Spurious transcription sequesters RNA polymerase molecules, hence reducing bacterial cell fitness. H-NS preventing intragenic transcription of AT-rich DNA would therefore avoid fitness costs (10, 11). H-NS modulates the expression of not only HGT DNA but also of core genes (12). H-NS consists of three structural domains: (i) a C-terminal domain, responsible for DNA binding (13); (ii) an N-terminal domain, responsible for dimerization (14–17); and (iii) a central dimer-dimer interaction domain, responsible for multimer formation (18, 19). In addition to homodimer and multimer formation, H-NS monomers are capable of heteromeric interactions with, among other proteins, members of the Hha family (20). These proteins show structural mimicry with the H-NS N-terminal domain and fine-tune the regulatory activity of H-NS-like proteins (12, 20, 21, 22).

The simultaneous presence of additional copies of *hns* homologues in the same cell is a relevant feature of this regulatory system. The enterobacterial genomes carry an *hns* paralogue, the *stpA* gene (23). The StpA protein is overexpressed in *hns* mutants (24). In *E. coli*, *stpA* mutants do not show a clear phenotype, and it has been suggested that StpA provides a molecular backup for H-NS in *E. coli*. In contrast, it has been shown that StpA modulates the expression of a significant number of genes in *Salmonella* (25). *hns* orthologues are also encoded by genes in plasmids (26). The IncHI1 plasmid pSF-R27 carries the *hns* orthologue *sfh*. Unlike H-NS, Sfh displays growth phase-dependent regulation (27). In the *Shigella flexneri* 2a strain 2457T, it was shown that each of the three proteins H-NS, StpA, and Sfh could form heterodimers with the corresponding homologues, thus suggesting that these proteins can modulate each other’s activities (27). Further studies showed that expression of the Sfh protein in cells harboring plasmid pSF-R27 provides a stealth function, avoiding the fact that plasmid incorporation results in a fitness cost for the bacterial host (28). The uropathogenic *E. coli* strain 536 contains, in addition to the *hns* and *stpA* genes, a third H-NS paralogue, the product of the *hfp* gene (29). The main regulatory role of the Hfp protein was found to occur at temperatures outside the host (25°C).

We report in this work the identification and characterization of a novel chromosomally encoded *hns* paralogue in the enteroaggregative *E. coli* (EAEC) strain 042 (open reading frame [ORF] EC042_2834). We present in this work experimental data showing that this variant has a specific role in modulating a subset of the H-NS-silenced genes.

## RESULTS

### **Identification of the H-NS paralogue ORF** EC042_2834** in the genome of the EAEC strain** 042.

The H-NS paralogue ORF EC042_2834 (from here on termed H-NS2) was identified in the annotated genome of *E. coli* strain 042 by performing a BLAST search (http://www.uniprot.org/blast/) using the amino acid sequence of the H-NS protein (UniProt accession no. D3H2L9) as the template. Figure S1 in the supplemental material shows the nucleotide and amino acid sequence alignments of H-NS and H-NS2.

10.1128/mSystems.00220-17.1 FIG S1  Alignment of H-NS and H-NS2 nucleotide and amino acid sequences. Alignments were performed using the T-Coffee algorithm (https://www.ebi.ac.uk/Tools/msa/tcoffee/). (A) Alignment of *hns* and *hns2* from *E. coli* 042 strain (locus tag EC042_1292 and EC042_2834, respectively). The nucleotides conserved in the two sequences are indicated by asterisks below the sequence alignment. (B) Alignment of H-NS and H-NS2 from *E. coli* 042 strain (protein_id WP_001287378.1 and WP_001278198.1, respectively). The conserved amino acid sequences are indicated by asterisks. Download FIG S1, TIF file, 0.2 MB. Copyright © 2018 Prieto et al. 2018 Prieto et al. This content is distributed under the terms of the Creative Commons Attribution 4.0 International license.

**FIG 1:**
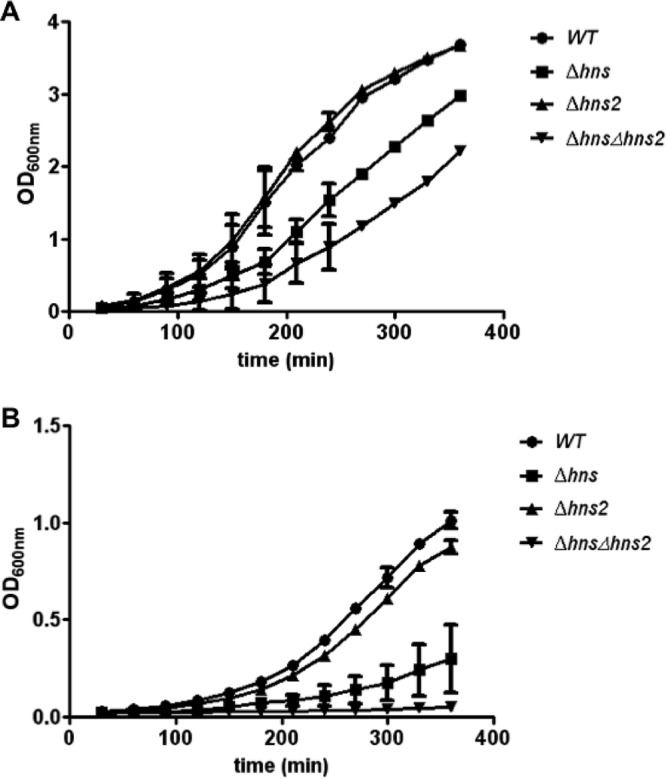
Impact of the *hns* and *hns2* alleles on growth of *E. coli* strain 042. (A and B) Growth kinetics of wild-type (WT) *E. coli* 042 and its *hns*, *hns2*, and *hns hns2* mutant derivatives in LB medium at 37°C (A) and 25°C (B). The experiments were performed in three biological replicates. Values are means ± standard deviations (error bars) are shown.

Upon identification of this new *hns* paralogue, we decided to obtain *hns*, *hns2*, and *hns hns2* mutant derivatives from wild-type (wt) EAEC strain 042 and then compared their growth rates at 37 and 25°C (Fig. 1A and B). At 37°C, the effect of the *hns2* allele on the growth rate is negligible. Nevertheless, the negative impact on the growth rate at 37°C of the *hns hns2* double mutant is higher than that of the *hns* mutant alone. At 25°C, the *hns2* allele alone moderately reduces the growth rate, and when it is combined with the *hns* allele, it drastically reduces the growth rate. These data suggest that, when strain 042 grows at 37°C, H-NS2 functions might be fulfilled by H-NS, but H-NS function could be only partially replaced by the existing H-NS2 protein levels. At low temperatures, H-NS2 function cannot be completely replaced by H-NS. Depletion of both proteins renders cells unable to grow at 25°C. We also studied whether the *hns* mutation by the *hns2* gene cloned in the vector pLG338-30 restores wt growth rate. *E. coli* MG1655 *hns* cells harboring the recombinant plasmid pLG338-30*hns2* show a growth rate at 37°C similar to that of wt cells (Fig. S2).

10.1128/mSystems.00220-17.2 FIG S2  H-NS2 protein complements an *hns* mutation in *E. coli* MG1655. Growth of *E. coli* strains MG1655 and its isogenic *hns* mutant derivative, either plasmid-free or harboring plasmid pLG388-30hns2. Download FIG S2, TIF file, 0.3 MB. Copyright © 2018 Prieto et al. 2018 Prieto et al. This content is distributed under the terms of the Creative Commons Attribution 4.0 International license.

### **Role of H-NS2 modulating gene expression in strain** 042.

We next studied the modulatory role of this novel H-NS paralogue. To do this, we decided to compare the effects of the *hns*, *hns2*, and *hns hns2* alleles on the transcriptome of strain 042. Taking into account the fact that several H-NS-modulated genes are comodulated by the Hha protein, we also studied the transcriptome of an *hha* mutant. As strain 042 contains two additional *hha* paralogues (*hha2* and *hha3*), to include one of these paralogues (*hha2*) in the modulation of gene expression together with *hha* (30), a *hha hha2* double mutant (considered an *hha* null mutant) was used instead of an *hha* single mutant. The complete results of transcriptome sequencing (RNA-seq) analyses are presented in Table S1. Taking into account the fact that the main role of H-NS or Hha is to silence gene expression, we analyzed in detail those genes that, compared with the wt strain, are upregulated in the different mutant genetic backgrounds (Table 1). The results obtained clearly show the existence of different groups of *E. coli* 042 genes with regard to Hha/H-NS/H-NS2 modulation: (i) genes upregulated in the *hha* null mutant that are also upregulated in the *hns* and *hns2* mutants (shown in red in Table 1) (remarkably, most of these genes are not upregulated in the *hns hns2* double mutant); (ii) genes that are highly upregulated in the *hns* and *hns hns2* mutants but are only modestly upregulated in the *hns2* mutant (shown in yellow in Table 1) (these genes are not upregulated in the *hha* null mutant); (iii) genes that are upregulated only in the *hns* mutant (shown in violet in Table 1). It is important to highlight that those genes showing the highest upregulation levels in the *hns2* mutant overlap with those that are upregulated in the *hha* null mutant. Hence, it is apparent that the H-NS2 protein has a specific role in repressing genes that require Hha for efficient silencing. The functions of those genes modulated by H-NS without requiring Hha are known in many instances, and several of these genes belong to the core genome (i.e., *hde* or *gad* operon). In contrast, the functions of most of the genes that are targeted by H-NS/Hha are not known. Most likely, they are HGT genes. The reported RNA-Seq data were also validated by quantitative reverse transcription-PCR (qRT-PCR) (Fig. S3).

10.1128/mSystems.00220-17.3 FIG S3  Confirmation by qRT-PCR of the transcriptomic data. Relative expression values of *gadB*, RS04580, and RS05870 genes in the *hns*, *hns2*, and *hns hns2* mutant derivatives of *E. coli* strain 042. Download FIG S3, TIF file, 0.1 MB. Copyright © 2018 Prieto et al. 2018 Prieto et al. This content is distributed under the terms of the Creative Commons Attribution 4.0 International license.

10.1128/mSystems.00220-17.7 TABLE S1  Summary of Illumina RNA-seq analysis containing reads and reads per kilobase per million (RPKM) values from *E. coli* 042 chromosome and plasmid pAA sequence (sense and antisense strand). Download TABLE S1, XLS file, 2 MB. Copyright © 2018 Prieto et al. 2018 Prieto et al. This content is distributed under the terms of the Creative Commons Attribution 4.0 International license.

**TABLE 1:**
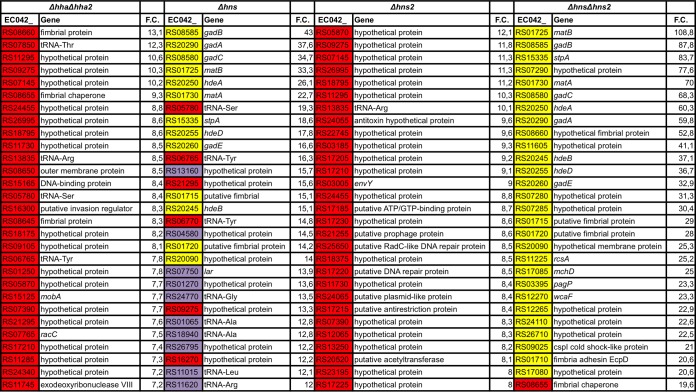
*E. coli* 042 genes showing highest upregulation levels in the different mutant backgrounds^a^

a The alleles or locus tags are shown in the *E. coli* 042 (EC042) columns. The genes or proteins are shown in the Gene columns. The fold change values are shown in the F.C. columns. The genes upregulated in the *hha* null mutant and in the *hns* and *hns2* mutants are shown in red. The genes that are highly upregulated in *hns* and *hns hns2* mutants and only modestly upregulated in the *hns2* mutant are shown in yellow. The genes showing upregulation only in the *hns* mutant are shown in violet. Note the coincidence between the set of genes upregulated in the *hha* null mutant and the *hns2* mutant. Whereas the functions of several genes shown in yellow are known, this is not the case for the genes shown in red (likely HGT genes).

### **Loss of H-NS or H-NS/H-NS2 function results in increased resistance to acid shock in strain** 042.

**FIG 2:**
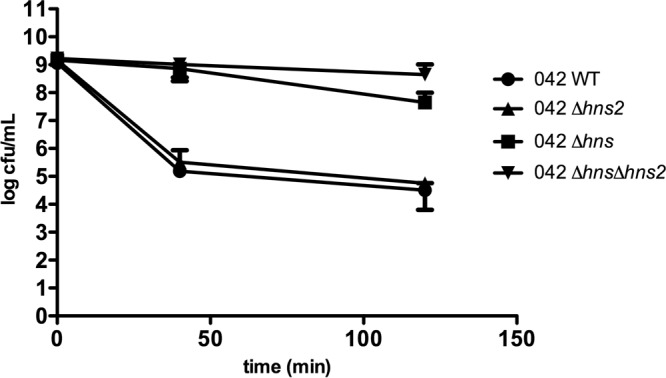
*hns* and *hns hns2* alleles result in increased acid shock resistance. Survival to acid shock of *E. coli* strain 042 and its *hns*, *hns2*, and *hns hns2* mutant derivatives. Experiments were performed in three biological replicates. Values are means ± standard deviations (error bars) are shown.

To correlate transcriptomic data with phenotype, we examined whether some of the observed deregulatory effects of both *hns* alleles on specific genes or operons could be correlated with predictable physiological changes. Taking into account the fact that genes in both the *gad* and *hde* operons participate in the adaptation of bacterial cells to acid pH, we studied survival of the wt strain, the *hns* and *hns2* single mutants, and the *hns hns2* double mutant under conditions of acid shock. The results obtained show that both the *hns* single mutant and the *hns hns2* double mutant are more resistant to acid shock (Fig. 2). These results are in accordance with the observed upregulation of *gad* and *hde* operons both in the *hns* single mutant and *hns hns2* double mutant.

### Regulation of the expression of the H-NS2 protein.

**FIG 3:**
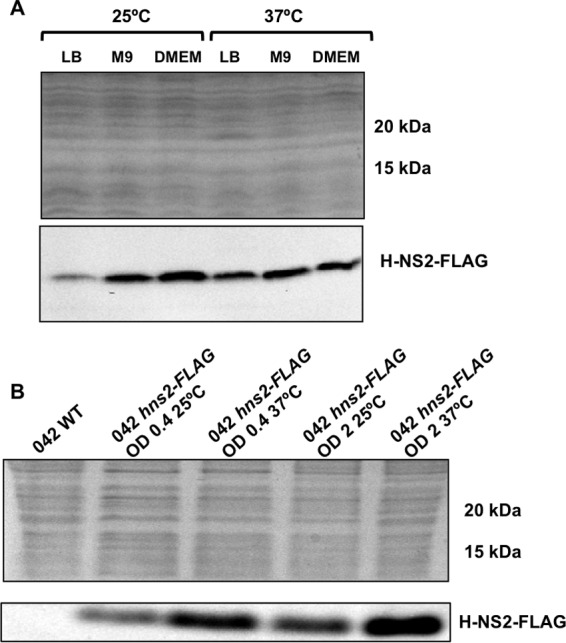
H-NS2-FLAG expression is upregulated in DMEM and M9 minimal medium and when cells enter the stationary growth phase. (A) Immunodetection of H-NS2-FLAG in cell extracts from *E. coli* strain 042 growing at 25°C and 37°C in LB, M9 minimal medium, and DMEM at the onset of the stationary phase (OD_600_ of 2.0). (B) Immunodetection of H-NS2-FLAG in cell extracts from *E. coli* strain 042 growing in LB medium at 25°C and 37°C both at the exponential and early stationary growth phases (OD_600 _of 0.4 and 2.0, respectively). Experiments were repeated three times. The results of a representative experiment are shown.

**FIG 4:**
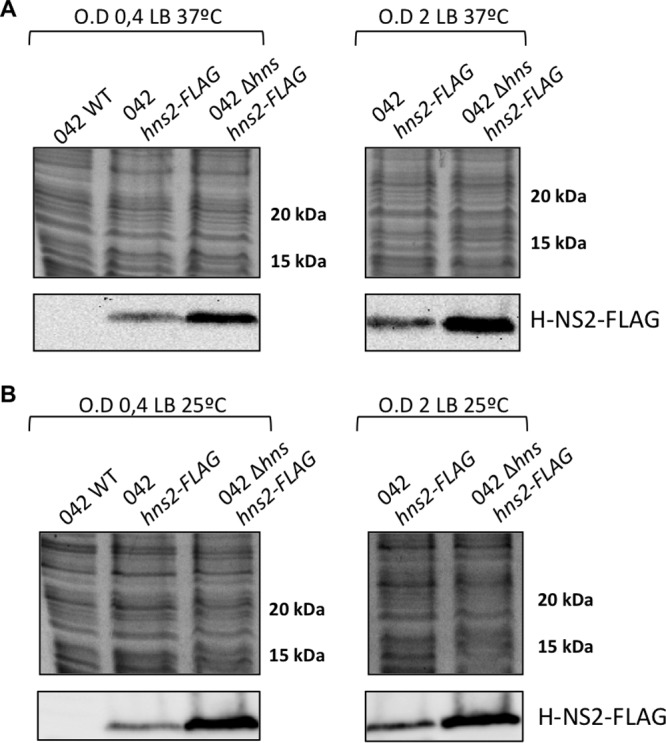
H-NS2 is upregulated in an *hns* mutant derivative of the strain *E. coli* 042. Immunodetection of H-NS2-FLAG in cultures of the wild-type (WT) *E. coli* 042 strain and its derivatives containing a FLAG tag in the *hns2* gene and an *hns* derivative containing a FLAG tag in the *hns2* gene. The top panels show Coomassie blue-stained cell extracts used for the immunodetection of H-NS2-FLAG. Similar protein concentrations are apparent. The bottom panels show immunodetected H-NS2-FLAG. The optical density at 600 nm (O.D) values are shown above the brackets.

It has been recently shown that the plasmid-encoded AraC negative regulator Aar modulates expression of both H-NS and H-NS2 in *E. coli* strain 042 (31). We decided to investigate whether, in addition to Aar, environmental or physiological factors influence H-NS2 expression. The *hns* promoter is known to be activated under conditions of cold shock (32) and is subjected to autoregulation (33–35). Out of these conditions, the H-NS levels are similar in cells growing under different conditions and also in cells collected at different stages of the growth curve (29, 36, 37). We studied whether the expression pattern of this novel variant is similar to that of H-NS. To compare expression of both H-NS proteins in strain 042, we decided to introduce a FLAG epitope and thereafter immunodetect them in cell extracts. Whereas the H-NS2-FLAG construct could be easily obtained, it was not possible to obtain the H-NS-FLAG construct. We assume that this modified H-NS variant is deleterious to 042 cells. We used the clone expressing H-NS2-FLAG to determine the effects of different growth conditions and physiological states on H-NS2 expression (Fig. 3). Compared with expression in rich medium (Luria broth [LB] medium), H-NS2 expression increases in cells growing in minimal medium and in Dulbecco modified Eagle medium (DMEM) (Fig. 3A). With respect to growth temperature, high temperature (37°C) increases H-NS2 expression in cells growing in LB medium (Fig. 3B). Western blotting data were also complemented with the results of qRT-PCR analysis of transcription of H-NS2, which confirmed the Western blotting data (Fig. S4). We analyzed H-NS expression by using H-NS-specific antibodies. As previously described (38), H-NS levels remained fairly constant in samples collected from cultures from strain 042 grown in the different conditions used (Fig. S5). We also determined H-NS2 expression in a strain 042 *hns* mutant in cells growing in LB medium at 25°C and 37°C. As expected, H-NS2 is overexpressed (Fig. 4).

10.1128/mSystems.00220-17.4 FIG S4  qRT-PCR analysis of *hns2* expression in *E. coli* 042 cells growing under different conditions. Relative expression values of the strain 042 *hns2* gene growing in different conditions. Expression in cells grown in LB medium at 37°C collected at an OD_600_ of 2.0 is arbitrarily set at 1. Download FIG S4, TIF file, 1.7 MB. Copyright © 2018 Prieto et al. 2018 Prieto et al. This content is distributed under the terms of the Creative Commons Attribution 4.0 International license.

10.1128/mSystems.00220-17.5 FIG S5  H-NS expression is not influenced by the temperature or the growth medium in *E. coli* 042. Immunodetection of H-NS with H-NS-specific antibodies in *E. coli* 042 cultures grown to the onset of the stationary phase (OD_600_ of 2.0) in LB, M9, and DMEM. Download FIG S5, TIF file, 0.1 MB. Copyright © 2018 Prieto et al. 2018 Prieto et al. This content is distributed under the terms of the Creative Commons Attribution 4.0 International license.

We used qRT-PCR to compare transcription of H-NS and H-NS2 proteins. Cells were grown in LB medium until the beginning of stationary phase (optical density at 600 nm [OD_600_] of 2.0), and transcripts were quantified. Transcription of the *hns* gene is several orders of magnitude higher (more than 80-fold) than transcription of the *hns2* gene.

### Interaction of H-NS2 with other proteins.

**FIG 5:**
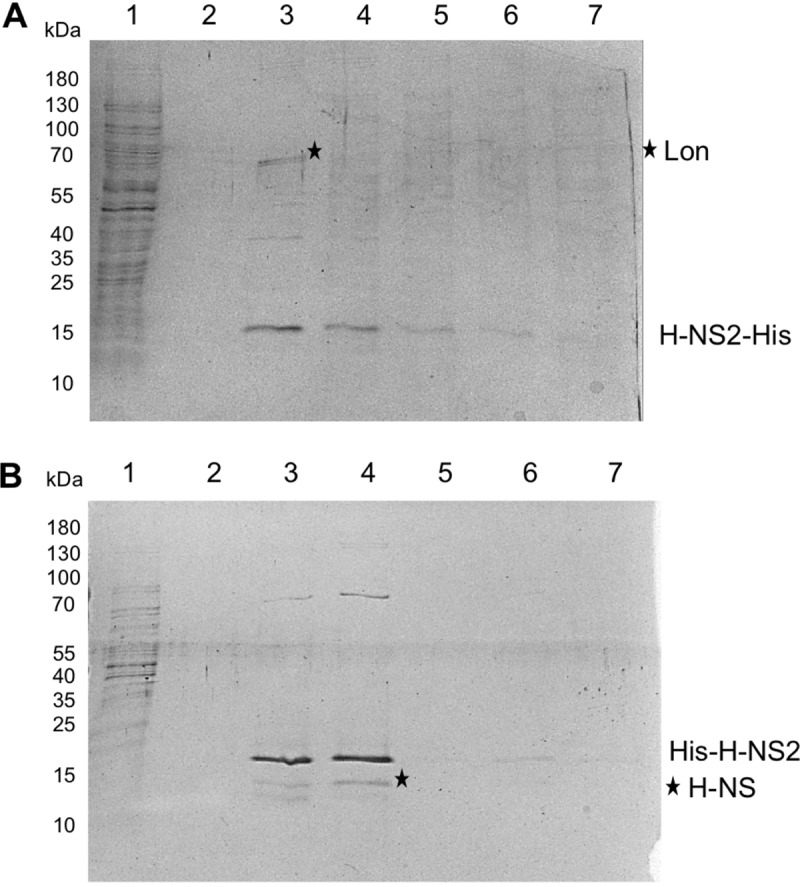
H-NS2 protein interacts with Lon and H-NS protein *in vitro*. (A and B) Analysis by SDS-PAGE and Coomassie blue staining of fractions from pulldown experiments after incubation of H-NS2 containing a His tag either at the C-terminal end (A) or N-terminal end (B) with a cellular extract from *E. coli* 042. Lanes 1 and 2 correspond to wash fractions with 50 mM imidazole. Lanes 3 to 5 correspond to elution fractions with 200 mM imidazole. Lane 6 corresponds to the elution fraction with 500 mM imidazole. Lane 7 corresponds to the elution fraction with 1 M imidazole. Bands indicated by black stars were identified by LC/MS-MS and correspond to Lon protein in panel A and H-NS protein in panel B. Experiments were repeated three times. The results of a representative experiment are shown.

**FIG 6:**
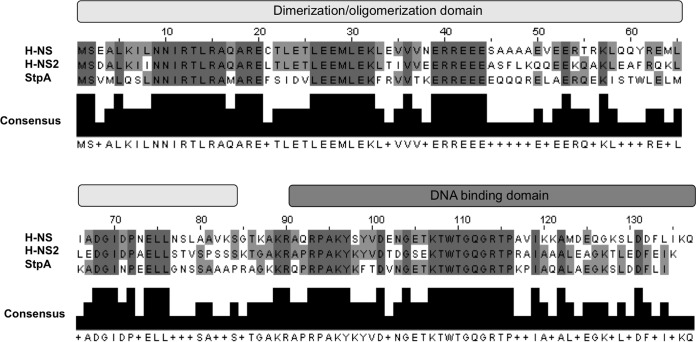
Alignment of H-NS, H-NS2, and StpA amino acid sequences. H-NS, H-NS2, and StpA sequence alignment showing the conserved amino acid sequence (dark and light gray). The black box shows the consensus sequence. Alignment was performed using the T-Coffee algorithm (https://www.ebi.ac.uk/Tools/msa/tcoffee/). The H-NS, H-NS2, and StpA sequences have been deposited in NCBI under accession numbers WP_001287378.1, WP_001278198.1, and WP_000115383.1, respectively. The light-gray and dark-gray bars above the sequence alignment show, respectively, the N- and C-terminal domains (dimerization/oligomerization and DNA binding domains, respectively).

Taking into account the fact that H-NS interacts, among other proteins, with StpA and Hha, we also studied the interaction of H-NS2 with other proteins. To do this, pulldown experiments were performed using His-tagged H-NS2 protein with the tag at the N- or C-terminal end. Two proteins could be identified as interacting with H-NS-2: H-NS and the Lon protease (Fig. 5). These results do not rule out an interaction with Hha. As the cellular concentration of Hha is not high, experimental evidence for copurification of Hha with His-tagged H-NS requires Hha overproduction (39). To confirm H-NS2 interaction with Hha, pulldown experiments were performed with His-tagged Hha protein. Liquid chromatography coupled to tandem mass spectrometry (LC-MS/MS) analysis of the fraction coeluting with Hha confirmed the presence of H-NS2 (Table S2). Because of the interaction of H-NS2 with Lon, we decided to ascertain whether, as shown for StpA (40), H-NS2 is sensitive to Lon-mediated proteolysis. In StpA, the amino acid residue at position 21 is F instead of C. In fact, the protease sensitivity would be lost after the mutation F21C (40). Interestingly, in H-NS2 protein, amino acid C is substituted by the hydrophobic amino acid L (Fig. 6). H-NS2 stability was measured in the wt strain and in *hns* and *lon* genetic backgrounds. No differences in stability could be found (Fig. S6).

10.1128/mSystems.00220-17.6 FIG S6  *In vivo* H-NS2 protein stability. Immunodetection of H-NS2-FLAG in samples collected from cultures grown at 37°C from strains 042 wt, 042*hns2*-Flag, 042*hns-hns2*-Flag, 042*lon-hns2*-Flag and 042*hnslon-hns2*-Flag. Samples were collected before (0 h) and 1 h and 2 h after the addition of chloramphenicol to the culture medium (LB). For a control of protein concentration in the cell extracts, the top panel shows Coomassie blue staining of the cell extracts. The bottom panel shows immunodetected H-NS2-FLAG. Download FIG S6, TIF file, 0.2 MB. Copyright © 2018 Prieto et al. 2018 Prieto et al. This content is distributed under the terms of the Creative Commons Attribution 4.0 International license.

10.1128/mSystems.00220-17.8 TABLE S2  LC-MS/MS analysis of the fraction coeluting with Hha in pulldown experiments performed with His-tagged Hha. Download TABLE S2, DOC file, 0.05 MB. Copyright © 2018 Prieto et al. 2018 Prieto et al. This content is distributed under the terms of the Creative Commons Attribution 4.0 International license.

### Distribution of the *hns2* gene within the *Enterobacteriaceae*.

**FIG 7:**
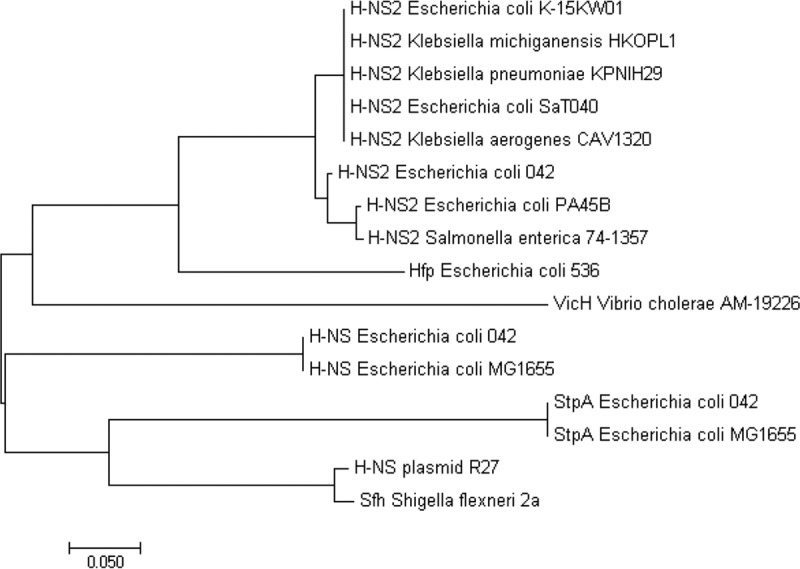
Evolutionary relationships of H-NS, StpA, and third H-NS paralogues in different members of *Enterobacteriaceae*. The dendrogram shows the relationships between the different proteins. Enterobacterial strains that contain a chromosomal copy of the *hns2* gene are presented. The amino acid sequences used from the following species are shown in the dendrogram (the NCBI accession numbers are shown in parentheses): *Klebsiella aerogenes* strain CAV1320 (AKK82112.1), *Klebsiella michiganensis* HKOPL1 (AHW87442.1), *Escherichia coli* strain K-15KW01 (ANR82355.1), *Escherichia coli* strain SaT040 (AML15501.1), *Klebsiella pneumoniae* subsp. *pneumoniae* strain KPNIH29 (AIX70716.1), *Escherichia coli* PA45B (ASW60759.1), *Salmonella enterica* serotype Enteritidis strain 74-1357 (ATT16728.1), *Escherichia coli* 042 H-NS (CBG35667), H-NS2 (CBG35667), and StpA (CBG35697), *Escherichia coli* MG1655 H-NS (NP_415753.1) and StpA (NP_417155.1), R27 plasmid H-NS (NP_058377.1), *Shigella flexneri* 2a Sfh (AAN38840.1), *Vibrio cholerae* AM-192226 VicH (ZP_04962344.1), and *Escherichia coli* 536 Hfp (ABG69928). The evolutionary history was inferred using the neighbor-joining method, and evolutionary analyses were conducted in MEGA7 (50). The bar shows 0.050 nucleotide substitutions per position.

We also studied the distribution of H-NS2 among members of the family *Enterobacteriaceae*. To assess this, we performed a BLAST search using the nucleotide sequence of the *hns2* gene as the template. The results obtained (Fig. 7) show that this novel *hns* paralogue is distributed in different genera of the *Enterobacteriaceae*, including, in addition to *Escherichia*, *Klebsiella* and *Salmonella*. All strains carrying the *hns2* gene also carried *hns* and *stpA*. The *hns2* sequence was identical in all strains. Interestingly, the third paralogue, Hfp, of *E. coli* 536 is closely related to H-NS2. This is not the case for the plasmid-encoded Sfh and H-NS_R27_ (the H-NS protein encoded by the IncHI plasmid R27 gene) proteins. We also aligned H-NS, H-NS2, and StpA amino acid sequences (Fig. 6). Overall, the H-NS2 sequence shows higher similarity to the H-NS sequence (64.4%) than to the StpA sequence (52.99%). When the dimerization/oligomerization domain (amino acid residues 1 to 83) and DNA binding domain (amino acids 91 to 137) are compared, H-NS2 shows higher similarity to H-NS than StpA in the dimerization/oligomerization domain (62.65% versus 53.01%) but lower similarity in the DNA binding domain (64.44% versus 70.5%).

## DISCUSSION

Several global regulators have been studied intensively in the last decades, and their biological role is well characterized. Nevertheless, this is not the case for their corresponding paralogues and orthologues, whose function remains obscure in many cases. Previous reports have shown that the cellular H-NS pool can include, in addition to the H-NS protein and its paralogue StpA, at least a third paralogue. This was reported for *Shigella flexneri* strain 2a 2457T, which harbors the pSF-R27 plasmid that expresses the Sfh protein (41), and for the uropathogenic *E. coli* strain 536, which expresses the Hfp protein (29). We show in this report that a new H-NS paralogue is expressed, among other enterobacterial strains, by EAEC strain 042. As expected from the already established biological role, the H-NS paralogues StpA or Hfp (23, 29), H-NS2 provides a molecular backup for H-NS. This can be shown when growth of wt 042 cells, *hns* or *hns2* single mutants, and *hns hns2* double mutant derivatives in LB medium is compared. The combination of *hns* and *hns2* alleles has a stronger impact in the growth rate than the *hns* allele alone.

A relevant difference between H-NS and H-NS2 is the set of genes targeted by these proteins. H-NS targets a large set of genes in strain 042, including both core genes and HGT genes. The latter set of genes is also modulated by Hha-like proteins, as shown in the transcriptomic analysis of the *hha* null mutant. Most of the genes showing high-level upregulation in an *hns* mutant (some of these genes are core genome genes) are only modestly upregulated in an *hns2* mutant and do not require comodulation by Hha. Examples are the genes belonging to the *gad*, *mat*, and *hde* operons. Interestingly, the most upregulated genes in an *hns2* mutant are the genes that are also the most upregulated in the *hha* null mutant. Hence, H-NS or H-NS2 modulates, with Hha, several *E. coli* 042 genes, most of them of unknown function and likely of HGT origin. In contrast, several core genes are mainly modulated by H-NS without the requirement for Hha. H-NS2 targeting a subset of the H-NS-modulated genes is also supported by the fact that some of the genes showing significant upregulation in an *hns* mutant show wt expression levels in the *hns2* mutant. When considering recent findings showing that a main role of H-NS is to silence transcription that occurs from intragenic promoters in AT-rich HGT DNA (10, 11), it can be hypothesized that H-NS2 participates in these processes in strain 042. It is also remarkable that, whereas most of the H-NS-modulated genes that are not targeted by Hha show the highest upregulation in a *hns hns2* double mutant, most of the Hha-modulated genes (which are comodulated by H-NS/H-NS2) show a wt regulatory pattern in that *hns hns2* mutant. In such a genetic background, the *stpA* gene is strongly upregulated. We hypothesize that high levels of StpA would occur when H-NS and H-NS2 are not available (avoiding unwanted upregulation of the set of genes modulated by the H-NS/Hha system).

H-NS2 preferentially targeting HGT genes is reminiscent of the role of the H-NS protein encoded by the IncHI plasmid R27 gene (H-NS_R27_) (12). H-NS_R27_ specifically silenced HGT genes, as Hha did. It is apparent that a global modulator needs to develop specific mechanisms to discriminate between different sets of genes to be regulated (i.e., HGT and core DNA). In the H-NS model, variants such as the plasmid gene-encoded H-NS_R27_ or the chromosomally encoded H-NS2 proteins appear to have evolved mainly to recognize structural domains of only a subset of the H-NS-modulated genes.

In addition to the already reported Aar-dependent modulation of H-NS2 (31), we show here that, unlike H-NS, H-NS2 shows temperature- and nutrient-dependent regulation. With respect to temperature, H-NS2 expression is higher at 37°C than at 25°C. This is consistent with H-NS2 modulating expression of strain 042 virulence determinants. With respect to nutrient concentration, it is apparent that a low growth rate (i.e., growth in mineral medium) results in higher H-NS2 expression. This may be correlated with growth within the host, conditions leading to reduced growth rates. Comparing *hns* and *hns2* transcription showed that *hns2* transcription is significantly lower than* hns* transcription. Because we were unable to obtain an H-NS-FLAG construct (most likely because the recombinant protein was deleterious), we could not directly compare H-NS and H-NS2 protein levels. Nevertheless, the significant difference in transcription predicts that H-NS2 levels would be significantly lower than H-NS levels. These data suggest that, whereas H-NS contributes simultaneously to chromosome architecture and gene silencing, H-NS2 displays only regulatory functions. The molecular mechanism by which proteins such as H-NS2 *in vivo* preferentially target some of the H-NS-regulated genes and not others remains to be elucidated. Band shift assays with different DNA fragments corresponding to the regulatory region of both subsets of H-NS-regulated genes did not show that H-NS2 preferentially binds to any of them, either in the presence or absence of the Hha protein (our unpublished results). In addition, no significant differences regarding curvature, AT percentage, or presence/absence of consensus H-NS binding sites could be identified by our *in silico* analysis of these DNA sequences (our unpublished data).

*In vitro* interaction of H-NS2 with Lon protease predicted that, as has been shown for StpA (40), H-NS2 may be subjected to Lon-mediated proteolysis. This assumption was reinforced by the fact that the cysteine 21 present in H-NS is replaced by the hydrophobic residues leucine in StpA and phenylalanine in H-NS2. Leucine 21 is required for StpA sensitivity to proteolytic cleavage (40). Nevertheless, testing of H-NS2 stability, both in the wt and in *hns* and *lon* mutants, did not provide evidence for H-NS2 being unstable. Whether H-NS2−Lon interaction is an artifact or indicates H-NS2 proteolytic degradation under conditions not tested by us remains to be elucidated.

According to the available information, the third H-NS paralogues share properties such as the following. (i) Their regulatory pattern can be different from that of H-NS. (ii) Their expression levels are in some instances lower than that of H-NS. (iii) They may expand and fine-tune the regulatory features of the H-NS system either by forming heteromeric complexes with H-NS/StpA or by recognizing only a subset of the H-NS-modulated genes (i.e., the genes modulated by the Hha family of proteins). The fact that all strains shown to possess the *hns2* gene also possess the *hns* and *stpA* genes suggests specific functions for the H-NS2 protein that are different from those of StpA. H-NS2 shows a higher degree of similarity to H-NS than to StpA. Interestingly, when considering the H-NS2 dimerization/oligomerization domain and DNA binding domain, the former shows a higher degree of similarity to H-NS than that of StpA, but the latter does not. This suggests H-NS2 showing dimerization/oligomerization properties more similar to those of H-NS, and differing more significantly in its DNA binding proteins. This can be correlated with the fact that several of the H-NS-targeted genes are not targeted by H-NS2. All strains found containing the *hns2* gene that have been characterized so far are virulent isolates displaying multiple antibiotic resistance phenotypes (42, 43). Further characterization of the role of the *hns2* gene in virulence regulation may contribute to developing specific strategies to combat infections caused by these strains.

## MATERIALS AND METHODS

### Bacterial strains, plasmids, and culture media.

All bacterial strains used in this work are listed in Table S3 in the supplemental material. Cultures were usually grown in Luria broth (LB) medium (10 g NaCl, 10 g tryptone, and 5 g yeast extract [all per liter]). Cultures were also grown in M9 minimal medium (44) and Dulbecco modified Eagle medium (DMEM) (Gibco) supplemented with 0.45% glucose with vigorous shaking at 200 rpm (Innova 3100 water bath shaker; New Brunswick Scientific). The following antibiotics were used at the concentrations indicated: kanamycin (50 μg ml^−1^), carbenicillin (100 μg ml^−1^).

10.1128/mSystems.00220-17.9 TABLE S3  Strains and plasmids used in this work. Download TABLE S3, DOC file, 0.1 MB. Copyright © 2018 Prieto et al. 2018 Prieto et al. This content is distributed under the terms of the Creative Commons Attribution 4.0 International license.

### Plasmid construction.

In order to perform complementation experiments, the *hns2* gene (open reading frame [ORF] EC042_2834) of *E. coli* 042 strain was cloned into the pLG338-30 vector. Primers hns2 pLG338 ECORI fw 5 (fw stands for forward) and hns2 pLG338 BAMHI rev 3 (rev stands for reverse) (see Table S4 for sequences) were used to PCR amplify the *hns2* gene using Phusion Hot Start II DNA polymerase (Thermo Fisher Scientific). The PCR fragment was purified using GeneJET PCR purification kit (Thermo Scientific) and digested with EcoRI and BamHI restriction enzymes (Thermo Scientific). Ligation was performed in pLG338-30 digested with the same restriction enzymes and treated with alkaline phosphatase. The resulting plasmid (pLG338-30*hns2*) was transformed into *E. coli* DH5α cells and selected in the presence of carbenicillin. For confirmation of correct in-frame insertion of the *hns2* gene, primers pLG338 EB Fw (Fw stands for forward) and pLG338 EB Rv (Rv stands for reverse) (see Table S4 for sequences) were used for sequencing.

10.1128/mSystems.00220-17.10 TABLE S4  Oligonucleotides used in this work. Download TABLE S4, DOC file, 0.1 MB. Copyright © 2018 Prieto et al. 2018 Prieto et al. This content is distributed under the terms of the Creative Commons Attribution 4.0 International license.

### Genetic manipulations.

All enzymes used to perform standard molecular and genetic procedures were used according to the manufacturer’s recommendations. To introduce plasmids in *E. coli*, bacterial cells were grown until an optical density at 600 nm (OD_600_) of 0.6 was reached. Cells were then washed several times with 10% glycerol, and the respective plasmids were introduced by electroporation using an Eppendorf gene pulser (Electroporator 2510).

Mutant derivatives lacking the *hns* and *hns2* genes in enteroaggregative *E. coli* (EAEC) strain 042 were obtained by the λ Red recombinant method (45). Briefly, the kanamycin antibiotic resistance cassette of plasmid pKD4 was amplified using oligonucleotides Hns042P1 and Hns042P2 and oligonucleotides 2834P1 and 2834P2 for *hns* and *hns2* deletions, respectively (see Table S4 for sequences). DNA templates were treated with DpnI (Thermo Fisher Scientific) following the manufacturer’s recommendations and then purified and electroporated to the competent cells. Mutants were selected on LB plates containing the appropriate selection marker (kanamycin in this case), and the successful deletion of the gene was confirmed by PCR using the KT primer (kanamycin resistance; Km^r^) in combination with specific primers located in the remaining gene sequence in the bacterial chromosome (see Table S4 for the sequence).

If necessary, the antibiotic resistance determinant was eliminated by transforming the mutant strain with plasmid pCP20 and subsequent incubation at 42°C for two or more passages as reported previously (45). The pCP20 plasmid carries the gene encoding the Flp recombinase that catalyzes recombination between the FRT sites flanking the kanamycin cassette (45). The double deletions were obtained by combining one previous deletion with another deletion associated with an antibiotic resistance cassette.

A chromosomal insertion of FLAG sequence into the *hns2* gene was obtained by a modification of the λ Red recombinant method, as previously described (46). The antibiotic resistance determinant of plasmid pSUB11 was amplified using oligonucleotides 28340423XP1 and 28340423XP2 (see Table S4 for sequences). Mutants were selected on LB plates containing kanamycin, and successful FLAG insertion was confirmed by PCR using the oligonucleotide KT (kanamycin resistance; Km^r^) in combination with specific oligonucleotides located in the remaining gene sequence nearby (28343XP1UP and 28343XP2DOWN, see Table S4 for sequences). The chromosomal fusion H-NS2-FLAG was constructed in the parental strain *E. coli* 042 and in the isogenic Δ*hns* mutant, generating 042*hns-hns2*-Flag.

### SDS-PAGE and Western blotting.

Whole-cell protein extracts (pelleted cells resuspended in Laemmli sample buffer) were separated on a 15% SDS-polyacrylamide gel. To detect specific proteins, they were transferred to a polyvinylidene difluoride (PVDF) membrane (Bio-Rad) by semidry electrotransfer. Prior to the Western blotting procedure, the total protein content of whole-cell extracts was checked by Coomassie blue staining.

For immunodetection of H-NS2-FLAG protein, a primary monoclonal anti-FLAG (Sigma) was used. To immunodetect H-NS native protein, a primary polyclonal anti-H-NS was used. Detection of primary antibodies bound to the specific proteins analyzed was performed with a horseradish peroxidase (HRP)-conjugated secondary antibody. The detection reagent used was ECL Prime Western blotting (GE Healthcare). Detection and the visualization of the chemiluminescent bands corresponding to the proteins being studied were performed using Molecular Imager ChemiDoc XRS system and Quantity One software (Bio-Rad).

### His tagging and pulldown experiment.

For overexpression of the H-NS2 protein, the *hns2* gene was cloned into the aLICator LIC cloning and expression vector (Thermo Fisher Scientific), following the manufacturer’s recommendations. The *hns2* gene was amplified by PCR using the Phusion Hot Start II DNA polymerase (Thermo Scientific) in combination with oligonucleotides (hns2_plate51NT fw and hns2_plate51NT rev) for N-terminal cloning and oligonucleotides (hns2_plate31CT fw and hns2_plate 31 CT rev) for C-terminal cloning. PCR products were purified using the GeneJET PCR purification kit (Thermo Scientific), and DNA concentration and quality were measured using a Nano-Drop 1000 instrument (Thermo Fisher Scientific). DNA ligations to pLATE vectors were performed following the manufacturer’s recommendations, generating plasmids pLATE51-6HisH-NS2 and pLATE31H-NS2-6His, respectively. His-tagged Hha (His-Hha) protein was purified as described (39). Pulldown experiments were performed using isopropyl-β-d-thiogalactoside (IPTG)-induced *E. coli* BL21(DE3) Δ*hns* cells containing plasmids pLATE31H-NS26His, pLATE516His H-NS2, and pET15bHisHha (Table S3). Cells were resuspended in A50 buffer (20 mM HEPES, 100 mM KCl, 5 mM MgCl_2_, and 50 mM imidazole), lysed by using a French press (three times at 800 lb/in^2^), and centrifuged. Supernatant-free cells containing overexpressed His-tagged H-NS2 and His-Hha proteins were puriﬁed using Ni^2+^-agarose resin (Qiagen). Briefly, Ni^2+^-agarose resin was washed ﬁve times with A50 buffer. Then, cell-free supernatant and Ni^2+^-agarose resin were mixed together and allowed to interact overnight at 4°C. After two washing steps of the resin with A50 buffer, His-tagged H-NS2 variants were eluted with the same buffer supplemented with 200 mM imidazole. Eluted proteins were analyzed by SDS-PAGE and stained with Coomassie blue to check the correct purification of the proteins. Afterward, the elution fractions were mixed with *E. coli* 042 total protein extract. Again, His-tagged H-NS2 and His-Hha proteins were newly purified. The proteins that copurified with H-NS2 and Hha were identified by liquid chromatography coupled to tandem mass spectrometry (LC-MS/MS).

### *In vivo* H-NS2 protein stability.

The intracellular stability of H-NS2-FLAG was evaluated as previously described (47). Protein stability was monitored after inhibition of protein synthesis by the addition of chloramphenicol to the corresponding bacterial cultures. Chloramphenicol (100 mg/ml) was added to bacterial cultures grown to an OD_600 _of 2.0 in LB medium at 37°C to a final concentration of 25 µg/ml. After antibiotic addition, samples were removed at the indicated time intervals and whole protein extracts were analyzed by Western blotting.

### Protein identification (LC-MS/MS).

Protein identification was performed at Proteomic Platform (Barcelona Science Park, Barcelona, Spain). Briefly, proteins were manually digested with trypsin (sequencing grade modified; Promega) in the gel. The excised band was washed sequentially with NH_4_HCO_3_ (25 mM) and acetonitrile (ACN). Proteins were reduced and alkylated by treatment with 20 mM dithiothreitol (DTT) solution for 60 min at 60°C, followed by treatment with a 50 mM solution of iodine acetamide for 30 min at room temperature, respectively. After sequential washings with buffer and acetonitrile, the proteins were digested overnight at 37°C with 200 ng of trypsin. Tryptic peptides were extracted from the gel matrix with 10% formic acid and acetonitrile; the extracts were pooled and dried in a vacuum centrifuge. The dried peptide mixture was analyzed in a nanoAcquity liquid chromatography column (Waters) coupled to an LTQ-Orbitrap Velos (Thermo Fisher Scientific) mass spectrometer. The tryptic digest was resuspended in 1% formic acid (FA) solution, and an aliquot was injected for chromatographic separation. Peptides were trapped on a Symmetry C18TM trap column (5 μm; 180 μm by 20 mm; Waters) and separated using a C_18_ reverse-phase capillary column (ACQUITY UPLC BEH column; 130 Å, 1.7 μm, 75 μm by 250 mm; Waters). The gradient used for the elution of the peptides was 1 to 40% solvent B in 30 min, followed by a gradient from 40% to 60% in 5 min (solvent A is 0.1% FA; solvent B is 100% ACN and 0.1% FA), with a 250 nl min^−1^ flow rate. Eluted peptides were subjected to electrospray ionization with an emitter needle (New Objective PicoTip; Scientific Instrument Services, Inc.) with an applied voltage of 2,000 V. Peptide masses (*m*/*z*, 300 to 1,700) were analyzed in data-dependent mode where a full-scan MS was acquired in the Orbitrap mass spectrometer with a resolution of 60,000 full width at half maximum (FWHM) at an *m*/*z* of 400. Up to the 15 most abundant peptides (minimum intensity of 500 counts) were selected from each MS scan and then fragmented in the linear ion trap using collision-induced dissociation (CID) (38% normalized collision energy) with helium as the collision gas. The scan time settings were as follows: 250 ms (1 microscan) for full MS and 120 ms for MSn. Generated .raw data files were collected with Thermo Xcalibur (v.2.2). The .raw file obtained in the mass spectrometry analysis was used to search against a database containing all entries for *Enterobacteriaceae* present in the public database UniProt (v.13/2/2017). A database containing common laboratory contaminant proteins was added to this database. The software used was Thermo Proteome Discoverer (v.1.4.1.14) with Sequest HT as the search engine. Both a target and a decoy database were searched in order to obtain a false-discovery rate (FDR), and thus estimate the number of incorrect peptide-spectrum matches that exceed a given threshold. The search results were visualized in Proteome Discoverer (v.1.4.1.14) and exported to Excel as a list of identified proteins.

### RNA-Seq.

RNA extraction, DNase treatment, and evaluation of RNA quality and cDNA libraries for Illumina sequencing were performed by Vertis Biotechnologie AG, Freising-Weihenstephan, Germany.

Total RNA was isolated from the cell pellets using a bead mill and the mirVana RNA isolation kit (Ambion) including DNase treatment. The total RNA preparations were examined by capillary electrophoresis. From the total RNA samples, rRNA molecules were depleted using the Ribo-Zero rRNA removal kit for bacteria (Illumina). From the rRNA-depleted RNA samples, first-strand cDNA was synthesized using an N6 randomized primer. After fragmentation, the Illumina TruSeq sequencing adapters were ligated in a strand-specific manner to the 5′ and 3′ ends of the cDNA fragments. The cDNA was finally amplified by PCR (15 PCR cycles) using a proofreading enzyme. For Illumina sequencing, cDNA libraries were pooled in a 25:1 ratio. The library pool was fractionated in the size range of 250 to 500 bp using a differential clean-up with the Agencourt AMPure kit. The cDNA pool was sequenced on an Illumina NextSeq 500 system using 75-bp read length. For single-end sequencing, we used an Illumina NextSeq 500 system and a MID 150 kit with a single 75-bp read length. Base calling was performed online during the sequencing procedure with the Real-Time Analysis (RTA) software version 2.4.11 and System Suite version 2.1.2.1. Illumina sequencing instruments generate per-cycle BCL base call files as primary sequencing output in the bcl2 format. Conversion of the bcl2 file to gzipped fastq files was performed using the bcl2fastq Script v. 2.18.0.12 provided by Illumina. Quality and adapter trimming was performed with the CLC Genomics Workbench 9.0 software package using the “Trim Sequences” tool with standard parameters. Mapping of the trimmed reads to the reference sequences was also performed with the CLC Genomics Workbench 9.0 using the “Map Reads to Reference” tool with standard parameters. For quantification of gene expression (read counting), the alignments generated with the Genomics Workbench were exported in BAM format. Read counting was then performed with the FeatureCounts v. 1.5.0-p1 program using the following parameters and settings: level, meta-feature level; paired-end, no; strand specific, yes; multimapping reads, counted (as fractions); multioverlapping reads, not counted; overlapping bases, 30; read orientations, fr.

### Quantitative reverse transcription-PCR (qRT-PCR).

Total RNA was isolated from bacterial pellets using the Tripure isolation reagent (Roche) according to the manufacturer’s recommendations. Potential traces of DNA were removed by digestion with DNase I (Turbo DNA-free; Ambion), according to the manufacturer’s instructions. RNA concentration and RNA quality were measured using a NanoDrop 1000 spectrophotometer (Thermo Fisher Scientific). For cDNA synthesis, 1 μg of total RNA isolated previously was reverse transcribed to generate cDNA using the High-capacity cDNA reverse-transcription kit (Applied Biosystems) according to the manufacturer’s protocol. All samples within an experiment were reverse transcribed at the same time, and the resulting cDNA was diluted 1:100 in nuclease-free water and stored in aliquots at −80°C until used. As a control, parallel samples were run in which reverse transcriptase was omitted from the reaction mixture. Real-time PCR was conducted using Maxima SYBR green/ROX qPCR master mix (2X) (Thermo Scientific) and the ABI Prism 7700 sequence detection system (Applied Biosystems). Specific oligonucleotides complementary to the genes of interest were designed using primer3 software (see Table S4 for sequence). Relative quantification of gene expression of mutants versus wild-type (wt) strain was performed using the comparative threshold cycle (*C*_*T*_) method (48). The relative amount of target cDNA was normalized using the *gapA* gene as an internal reference standard. Fold change values referring to relative expression of target genes in mutant strains versus the wt strain were calculated by dividing the Δ*C*_*T*_ (difference between the *C*_*T*_ values for the target gene and the internal reference standard *gapA* gene) obtained for the different mutant strains versus the wt strain.

### Acid shock assay.

The acid shock assay was performed as described previously (49). Briefly, bacterial cultures were grown in LB medium to early stationary phase (OD_600 _of 2.0) and subjected to acid stress by adding gradually 6 N HCl to cultures until a pH of 3.2 was reached. The pH values were monitored by pH measurements on a separate culture. After acid addition, the cultures were shaken at 37°C for 30 min, 1 h, and 2 h. At the corresponding time intervals, cells were serially diluted in 0.9% NaCl and then plated on LB agar plates for colony counting.

### H-NS2 phylogeny.

*hns* paralogues were identified by performing a BLAST search (blast.ncbi.nlm.nih.gov/Blast.cgi) using the nucleotide sequence of the *hns* gene from *E. coli* 042 strain (ORF EC042_1292) as the template. Then, a phylogenetic tree with H-NS, StpA, and third H-NS paralogues were constructed using neighbor joining as a clustering method conducted in MEGA7 (50).

### Accession number(s).

The RNA sequencing reads have been deposited in the Gene Expression Omnibus (GEO) Sequence Read Archive of the National Center for Biotechnology Information (GSE105133) under accession numbers GSM2822965, GSM2822966, GSM2822967, GSM2822968, and GSM2822969.
