# Supplementary figures and images for: Evolution of Bacterial Global Modulators: Role of a Novel H-NS Paralogue in the Enteroaggregative Escherichia coli Strain 042
Source: mSystems. 2018 Mar 20;3(3):e00220-17. doi: 10.1128/mSystems.00220-17 (PMC5861252; doi:10.1128/mSystems.00220-17)

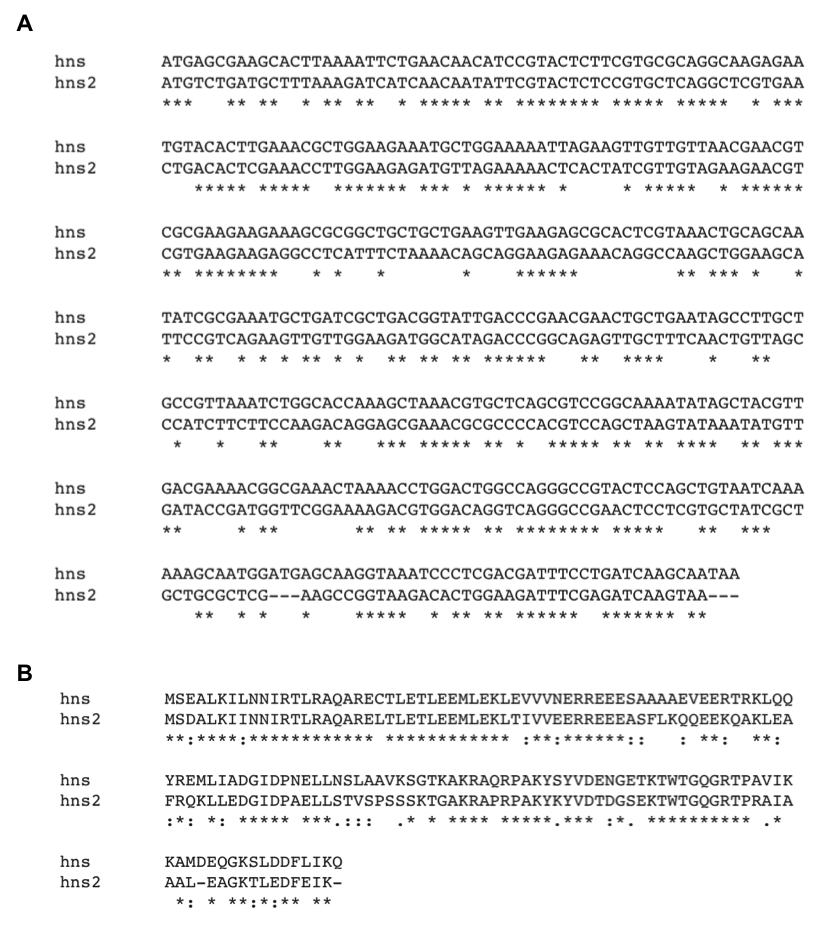

Supplement: FIG S1 [file sys001182204sf1.tif]

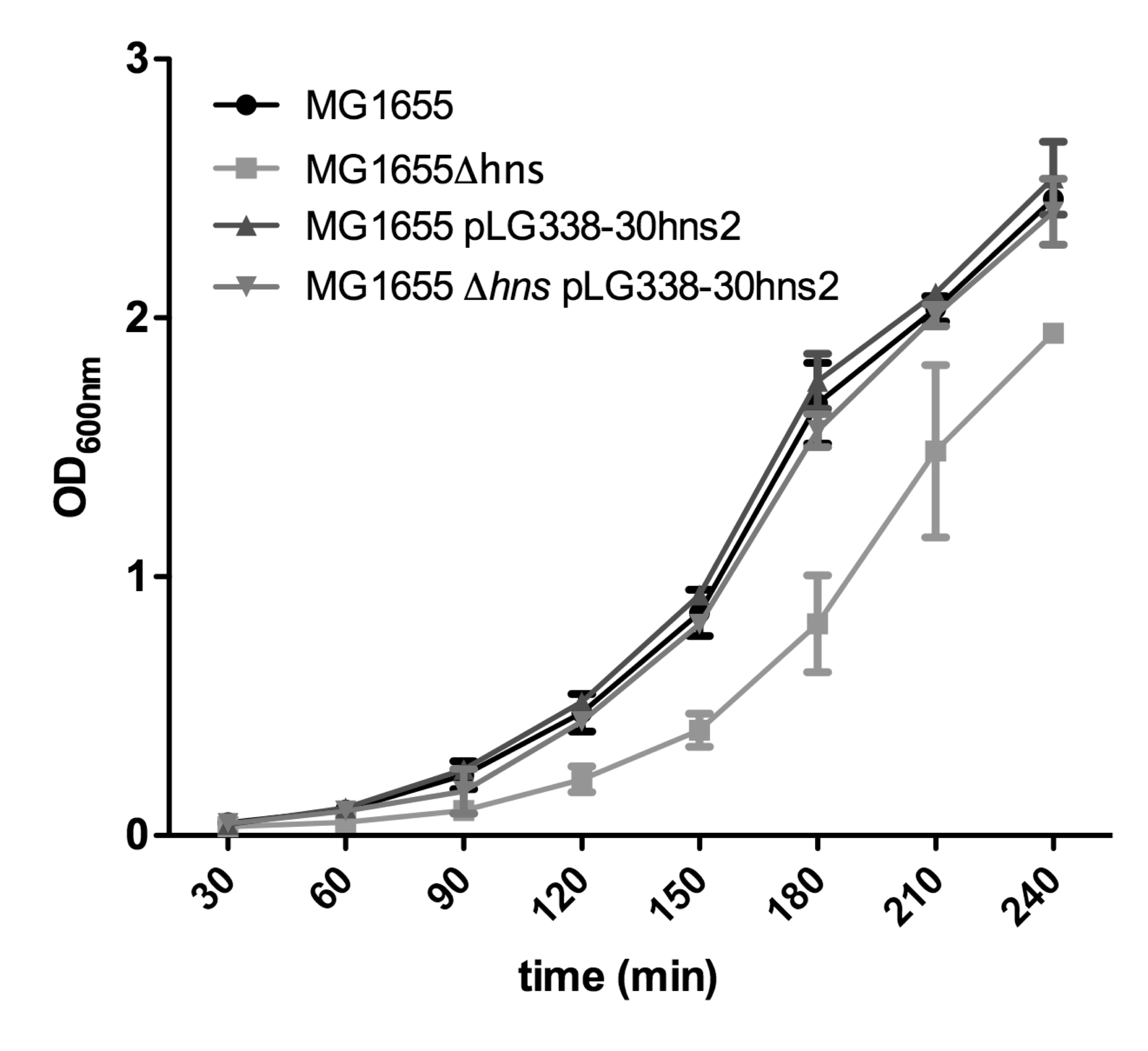

Supplement: FIG S2 [file sys001182204sf2.tif]

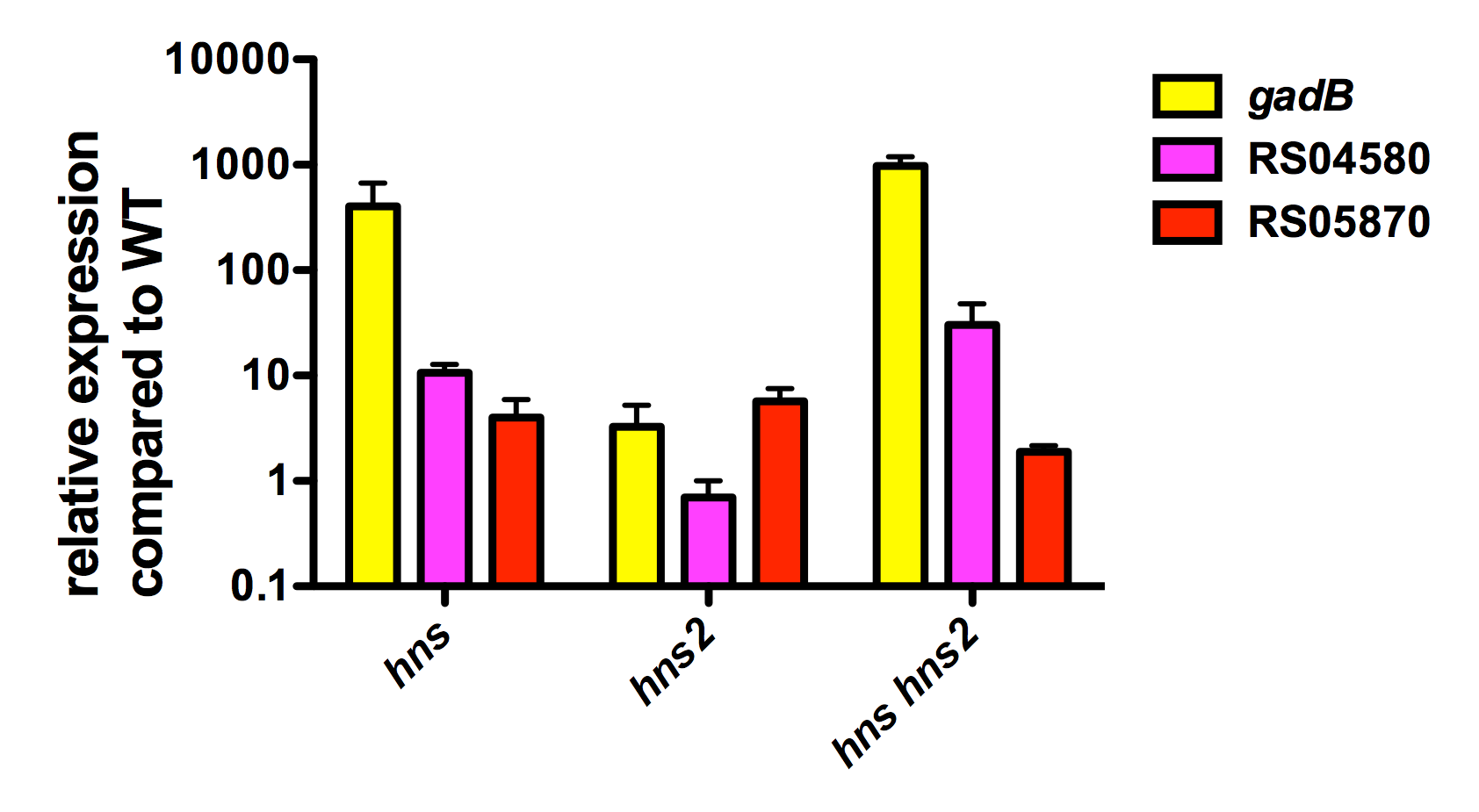

Supplement: FIG S3 [file sys001182204sf3.tif]

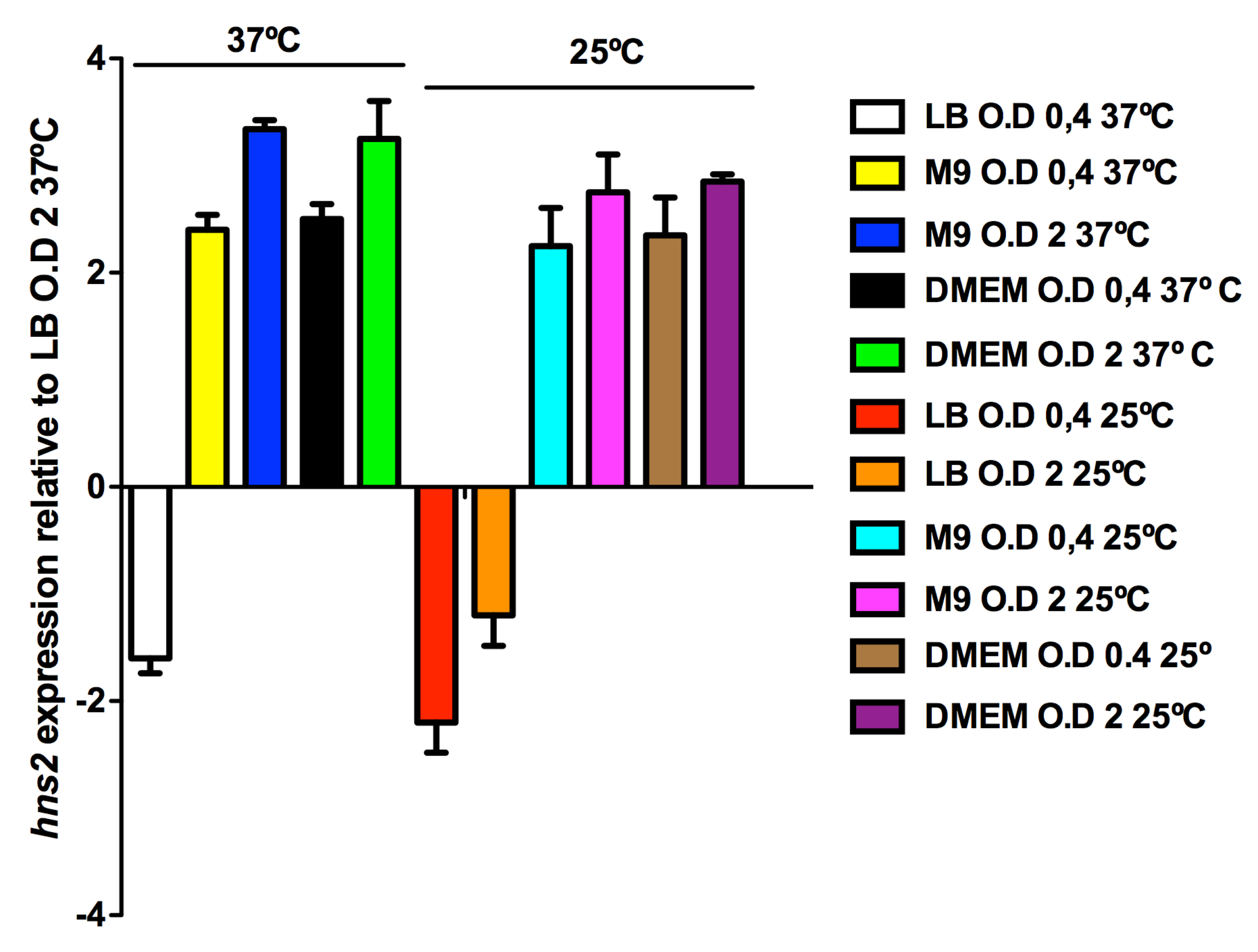

Supplement: FIG S4 [file sys001182204sf4.tif]

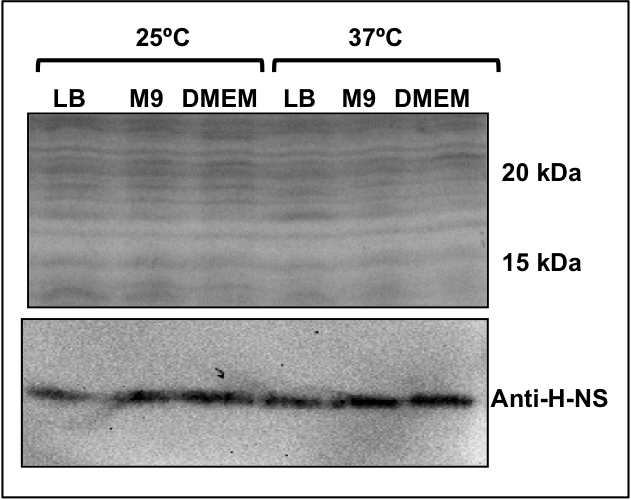

Supplement: FIG S5 [file sys001182204sf5.tif]

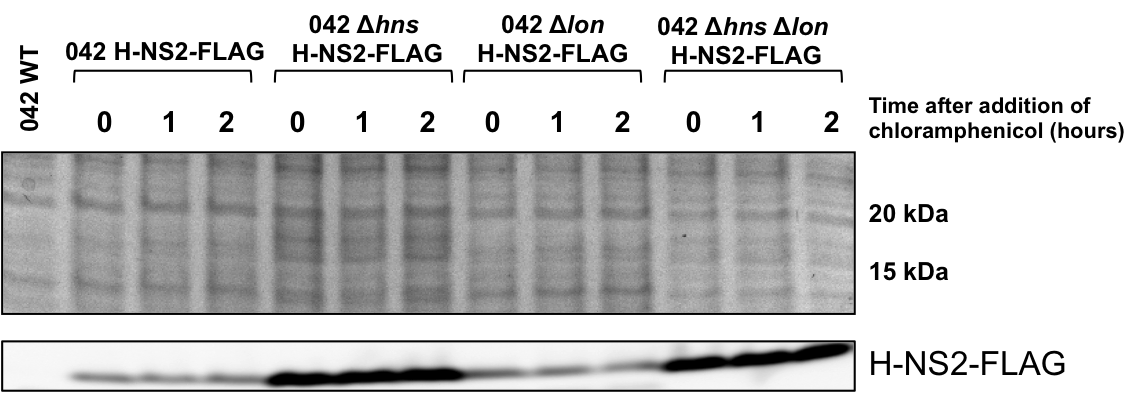

Supplement: FIG S6 [file sys001182204sf6.tif]
