# Supplementary material for: Evolution of Bacterial Global Modulators: Role of a Novel H-NS Paralogue in the Enteroaggregative Escherichia coli Strain 042
Source: mSystems. 2018 Mar 20;3(3):e00220-17. doi: 10.1128/mSystems.00220-17 (PMC5861252; doi:10.1128/mSystems.00220-17)
Supplement: TABLE S2 [file sys001182204st2.doc]

| **NCBI – ID** | **Protein name** | **Score** | **Coverage** | **Proteins** | **Unique Peptides** | **Peptides** | **PSMs** | **AAs** | **MW [kDa]** | **calc. pI** |
| --- | --- | --- | --- | --- | --- | --- | --- | --- | --- | --- |
| 16759453 | hemolysin expression-modulating protein [Salmonella enterica subsp. enterica serovar Typhi str. CT18] - **Hha** | 315,73 | 86,11 | 10 | 11 | 11 | 93 | 72 | 8,6 | 8,72 |
| 419925366 | global DNA-binding transcriptional dual regulator H-NS [Escherichia coli 541-15] – **H-NS** | 220,91 | 59,85 | 2 | 8 | 9 | 59 | 137 | 15,5 | 5,47 |
| 309786266 | DNA-binding protein stpA [Shigella dysenteriae 1617] - **StpA** | 83,15 | 53,39 | 6 | 5 | 5 | 26 | 118 | 13,5 | 5,72 |
| 215489283 | glycerol dehydrogenase [Escherichia coli O127:H6 str. E2348/69] | 80,13 | 58,04 | 7 | 10 | 10 | 21 | 367 | 38,7 | 4,88 |
| 387608280 | putative histone-like DNA-binding protein [Escherichia coli 042] – **H-NS2** | 55,97 | 50,37 | 1 | 6 | 7 | 17 | 135 | 15,2 | 5,47 |
| 110644322 | DNA-directed RNA polymerase subunit beta [Escherichia coli 536] | 51,58 | 12,07 | 31 | 11 | 11 | 16 | 1342 | 150,6 | 5,26 |
| 419415408 | DNA-directed RNA polymerase, beta' subunit [Escherichia coli DEC15E] | 41,68 | 12,69 | 19 | 10 | 10 | 14 | 1316 | 144,6 | 6,58 |
| 432543769 | transcriptional regulator [Escherichia coli KTE236] | 38,68 | 23,51 | 3 | 4 | 4 | 10 | 268 | 30,0 | 6,44 |
| 15800475 | phosphotransferase [Escherichia coli O157:H7 str. EDL933] | 37,95 | 44,49 | 30 | 7 | 7 | 13 | 272 | 30,2 | 5,77 |
| 190341311 | RpoA [Enterobacter aerogenes] | 36,50 | 50,00 | 12 | 10 | 10 | 14 | 308 | 34,1 | 5,20 |
| 297521513 | pyruvate dehydrogenase subunit E1 [Escherichia coli OP50] | 31,82 | 8,60 | 27 | 4 | 4 | 8 | 698 | 78,8 | 5,76 |
| 417130849 | formate C-acetyltransferase [Escherichia coli 5.0588] | 21,56 | 13,74 | 9 | 6 | 6 | 6 | 728 | 81,6 | 5,83 |

**Table S2.** LC-MS/MS analysis of the fraction co-eluting with Hha in pull-down experiments performed with His-tagged Hha.
