# Supplementary material for: Evolution of Bacterial Global Modulators: Role of a Novel H-NS Paralogue in the Enteroaggregative Escherichia coli Strain 042
Source: mSystems. 2018 Mar 20;3(3):e00220-17. doi: 10.1128/mSystems.00220-17 (PMC5861252; doi:10.1128/mSystems.00220-17)
Supplement: TABLE S3 [file sys001182204st3.doc]

| **Bacterial strains** | **Description** | **Source or reference(s)** |
| --- | --- | --- |
| 042 | *E. coli* EAEC, Cm^r^ Sm^r^ Tc^r^ | Prof. I. Henderson |
| 042*hns* | 042 derivative carrying *hns* (EC042_1292) deletion | (1) |
| 042*hns2* | 042 derivative carrying *hns2* (EC042_2834) deletion | This work |
| 042*hnshns2* | 042 derivative carrying *hns and hns2* deletion | This work |
| 042*hns2*-Flag | Epitope 3XFLAG added to *hns2* C-terminal | This work |
| 042*hns*-*hns2*-Flag | 042*hns* and Epitope 3XFLAG added to *hns2* C-terminal | This work |
| 042*lon*-*hns2*-Flag | 042*lon* and Epitope 3XFLAG added to *hns2* C-terminal | This work |
| 042*hnslon*-*hns2*-Flag | 042*hns lon* and Epitope 3XFLAG added to *hns2* C-terminal | This work |
| 042*hhahha2* | 042 derivative carrying *hha* (EC042_0498) and *hha2* (EC042_4516) deletions | (2) |
| MG1655 | *E. coli* K12 substr. MG1655 | Lab stock |
| MG1655H | MG1655 Δ*hns* | (1) |
| BL21 (DE3) Δ*hns* | BL21 (DE3) Δ*hns*::Km | (3) |
| DH5α | *Φ80dlacZΔM15 recA1 endA1 gyrA96 thi1 hsdR17 (rk- mk+) supE44 relA1 deoR Δ(lacZYA-argF)U169 phoA* | (4) |
| **Plasmids** | **Description** | **Source or reference(s)** |
| pKD4 | oriRγ, Km^r^, Ap^r^ | (5) |
| pKD46 | oriR101, repA101 (ts), AraBp-gam-bet-exo | (5) |
| pSUB11 | -FLAG and -Km^r^ coding template vector | (6) |
| pCP20 | λcI857 (ts), ts-rep (Recombinase FLP ts) | (5) |
| pLATE51 | Vector for overexpress and His-tag protein at N-terminal. | Thermo Scientific |
| pLATE51-6HisH-NS2 | pLATE51 + *hns2* gene from *E. coli* 042 (EC042_2834) | This work |
| pLATE31 | Vector for overexpress and His-tag protein at C-terminal. | Thermo Scientific |
| pLATE31H-NS2-6His | pLATE31 + *hns2* gene from *E. coli* 042 (EC042_2834) | This work |
| pLG338-30 | Ori_pSC101_, Ap^r^ | (7) |
| pLG338-30*hns2* | pLG338-30 + *hns2* gene from *E. coli* 042 (EC042_2834) | This work |
| pET15bHisHha | pET15b + 6xhis-*hha* Ap^r^ | (8) |

**Table S6.** Strains and plasmids used in this work.

**References**

1. **Hüttener M**, **Dietrich M**, **Paytubi S**, **Juárez A**. 2014. HilA-like regulators in *Escherichia coli* pathotypes: the YgeH protein from the enteroaggregative strain 042. BMC Microbiol **14**:268.

2. **Prieto A**, **Urcola I**, **Blanco J**, **Dahbi G**, **Muniesa M**, **Quirós P**, **Falgenhauer L**, **Chakraborty T**, **Hüttener M**, **Juárez A**. 2016. Tracking bacterial virulence: global modulators as indicators. Sci Rep **6**:25973.

3. **Zhang A**, **Rimsky S**, **Reaban ME**, **Buc H**, **Belfort M**. 1996. *Escherichia coli* protein analogs StpA and H-NS: regulatory loops, similar and disparate effects on nucleic acid dynamics. EMBO J **15**:1340–1349.

4. **Taylor RG**, **Walker DC**, **McInnes RR**. 1993. *E. coli* host strains significantly affect the quality of small scale plasmid DNA preparations used for sequencing. Nucleic Acids Res **21**:1677–1678.

5. **Datsenko KA**, **Wanner BL**. 2000. One-step inactivation of chromosomal genes in *Escherichia coli* K-12 using PCR products. Proc Natl Acad Sci USA **97**:6640–6645.

6. **Uzzau S**, **Figueroa-Bossi N**, **Rubino S**, **Bossi L**. 2001. Epitope tagging of chromosomal genes in *Salmonella.* Proc Natl Acad Sci USA **98**:15264–15269.

7. **Cunningham TP**, **Montelaro RC**, **Rushlow KE**. 1993. Lentivirus envelope sequences and pro viral genomes are stabilized in *Escherichia coli* when cloned in low-copy-number plasmid vectors. Gene **124**:93–98.

8. **Cordeiro TN**, **García J**, **Pons J-I**, **Aznar S**, **Juárez A**, **Pons M**. 2008. A single residue mutation in Hha preserving structure and binding to H-NS results in loss of H-NS mediated gene repression properties. FEBS Lett **582**:3139–3144.
