# Supplementary material for: Evolution of Bacterial Global Modulators: Role of a Novel H-NS Paralogue in the Enteroaggregative Escherichia coli Strain 042
Source: mSystems. 2018 Mar 20;3(3):e00220-17. doi: 10.1128/mSystems.00220-17 (PMC5861252; doi:10.1128/mSystems.00220-17)
Supplement: TABLE S4 [file sys001182204st4.doc]

| **Oligonucleotide name** | **Sequence (5’-3’)** | **Use** |
| --- | --- | --- |
| hns p1 | ATGAGCGAAGCACTTAAAATTCTGAACAACATCCGTACTCTTGTGTAGGCTGGAGCTGCTTC | *hns* deletion |
| hns p2 | TTATTGCTTGATCAGGAAATCGTCGAGGGATTTACCTTGCTGCATATGAATATCCTCCTTAGT | *hns* deletion |
| hns p1 up | CCTCACGTGCTGCGAAATCA | *hns* deletion confirmation |
| hns p2 down | GCAGGCCTTCGTTGAATACG | *hns* deletion confirmation |
| hns2 p1 | GCTTGTATTCTTTACCACAAACATTAGGGAAATCGCATGTCTGTGTAGGCTGGAGCTGCTTC | *hns2* deletion |
| hns 2 p2 | AAAATGAGCGGATTTGGTAGACATATGGTATGAAGATTACTTCATATGAATATCCTCCTTAGT | *hns2* deletion |
| hns2 p1 up | CGGCAATACAATCGAATACT | *hns2* deletion and FLAG confirmation |
| hns2 p2 down | CGTTTGTAAGTCCCGTGTGC | *hns2* deletion and FLAG confirmation |
| lon p1 | TACCTGGCGGAAATTAAACTAAGAGAGAGCTCTATGAATCCTGTGTAGGCTGGAGCTGCTTC | *lon* deletion |
| lon p2 | CCTGTTTTTATTAGTGCATTTTGCGCGAGGTCACTATTTTGCCATATGAATATCCTCCTTAGT | *lon* deletion |
| lon p1 up | CTATTCTCGGCGTTGAATGTG | *lon* deletion confirmation |
| lon p2down | TACAACACCCCTTAATAAGGG | *lon* deletion confirmation |
| hns23x p1 | GCTCGAAGCCGGTAAGACACTGGAAGATTTCGAGATCAAGGACTACAAAGACCATGACGG | *hns2* FLAG insertion |
| hns23x p2 | ATGAGCGGATTTGGTAGACATATGGTATGAAGATTACTTGCATATGAATATCCTCCTTAG | *hns2* FLAG insertion |
| KT | CGGCCACAGTCGATGAATCC | Km cassette confirmation |
| hns2_plate51NT fw | GGTGATGATGATGACAAGATGTCTGATGCTTTAAAGATCATCAACAATATTCGT | Cloning  H-NS2 |
| hns2_plate51NT rev | GGAGATGGGAAGTCATTACTTGATCTCGAAATCTTCCAGTGTCTTACCGGC | Cloning  H-NS2 |
| hns2_plate31CT fw | AGAAGGAGATATAACTATGTCTGATGCTTTAAAGATCATCAACAATATTCGTACT | Cloning  H-NS2 |
| hns2_plate 31 CT rev | GTGGTGGTGATGGTGATGGCCCTTGATCTCGAAATCTTCCAGTGTCTTACCGGC | Cloning  H-NS2 |
| hns2 pLG338 ECORI fw 5 | CGGAATTCTAAATTTGAACTCAGGATGGA | Cloning *hns2* |
| hns2 pLG338 BAMHI rev 3 | CGGGATCCTGGCATGTTAATCCCTGTAGG | Cloning *hns2* |
| pLG338 EB Fw | CCGCGCACATTTCCCCGAAAA | Confirmation cloning into pLG338-30 vector |
| pLG338 EB Rv | GCCAGTGCCAAGCTAGGCCTA | Confirmation cloning into pLG338-30 vector |
| gapAFW | TTTCCGTGCTGCTCAGAAAC | qPCR |
| gapARV | TTTCCGTGCTGCTCAGAAAC | qPCR |
| gadBFW | GGTATAGCCTTCACGACCGA | qPCR |
| gadBRV | CGCGTGTGAAATCGATCAGT | qPCR |
| RS04580FW | TTGAGTCCCTGGTTCCTGAG | qPCR |
| RS04580RV | CACATCATGCCCGTTGTTATT | qPCR |
| RS05870FW | CTTTAGGAGAAGCACGGAAAGT | qPCR |
| RS05870RV | CAGCCATTCCGACAACAGAA | qPCR |
| hns RT Fw | TGCGCAGGCAAGAGAATGTA | qPCR |
| hns RT Rv | AGCAGTTCGTTCGGGTCAAT | qPCR |
| hns2 RT Fw | TCGGAAAAGACGTGGACAGG | qPCR |
| hns2 RT Rv | AGTGTCTTACCGGCTTCGAG | qPCR |

**Table S4.** Oligonucleotides used in this work.
